# Rhinitis, sinusitis and ocular disease – 2085. Prevalence of allergic rhinitis in urban school children, Jaipur City, India

**DOI:** 10.1186/1939-4551-6-S1-P164

**Published:** 2013-04-23

**Authors:** Abhishek Saini, Mukesh Gupta, Bhagwan Sahai Sharma, Munish Kakkar, Gaurav Chaturvedy, Mukesh Gupta

**Affiliations:** 1 Mahatma Gandhi Medical & Technical University, Sitapura, Jaipur, India; 2 SMS Medical College, Jaipur, Jaipur, India; 3 Paed, MGH, India

## Background

The present study is conducted to find the prevalence of Allergic Rhinitis (AR) and its associated co-morbidity in school going children in urban area of Jaipur City, Rajasthan.

## Methods

A questionnaire based survey was conducted during November-December 2011 in 2300 school children (4-18 yr). Modified ARIA questionnaire translated in Hindi language containing 20 questions was used. Questions 1-4 were AR defining symptoms, question 5 related to classification (intermittent Vs persistent), questions 6 related to allergens and conditions inducing or aggravating symptoms and question 7-20 were related to co morbidities and family history. We screened questionnaires and those who answered yes to any AR defining symptoms were shortlisted. Children having two or more rhinitis symptoms (blocked nose, running nose, sneezing and nasal itching) in association with minimum 2 identifiable triggers were considered to have allergic rhinitis. Detailed history and physical examination was done by team consisting a pediatrician and ENT specialist in cases wherever diagnosis was in doubt.

## Results

Out of 2300 questionnaire forms distributed, 1693 were returned (response rate 73.60%) and 1572 forms were adequately filled (92.85%). Prevalence of AR in different age groups was as follows: 4-8 years 33.87% (167/493), 8-12years 34.87% (143/410) and in 12-18 years 32.43 % (217/669) with an overall prevalence 33.52% (527/1572). Male: Female of the cohort was 1.9:1. Nasal obstruction was the most frequent symptom (n=256, 48.57 %) followed by running nose (n=174, 33.01%), sneezing (n=127, 24.09%) and nasal itching (n=107, 20.30%). Of these 527 children with AR 35.67% (188/527) children had intermittent symptoms while 64.33% (339/527) had persistent rhinitis. Majority of the children with AR (306/527, 58.06%) had one or more co-morbidity while 116 (22.01%) had 2 or more co –morbidities and only 221 children (41.93%) children had AR without any co morbidity. The prevalence of different co morbidities was as follows: Adenoids 119 (22.5%), asthma 101 (19.16%), recurrent or chronic otitis media 125 (23.71%), sleep disturbances in 48 (9.1%), conjunctivitis 41 (7.78%) and laryngitis in 06 (1.13%).

## Conclusions

A high prevalence of AR and its co morbidities was observed in school children as compared to previous reports from this area.

